# Cytogenetic studies in *Eigenmannia virescens *(Sternopygidae, Gymnotiformes) and new inferences on the origin of sex chromosomes in the *Eigenmannia *genus

**DOI:** 10.1186/1471-2156-10-74

**Published:** 2009-11-21

**Authors:** Danillo S Silva, Susana SR Milhomem, Julio C Pieczarka, Cleusa Y Nagamachi

**Affiliations:** 1 Laboratório de Citogenética, Instituto de Ciências Biológicas, Universidade Federal do Pará, Avenida Perimetral, sn Guamá. Belém, Pará, 66075-900, Brazil

## Abstract

**Background:**

Cytogenetic studies were carried out on samples of *Eigenmannia virescens *(Sternopygidae, Gymnotiformes) obtained from four river systems of the Eastern Amazon region (Para, Brazil).

**Results:**

All four populations had 2n = 38, with ZZ/ZW sex chromosomes (Z, acrocentric; W, submetacentric). Constitutive heterochromatin (CH) was found at the centromeric regions of all chromosomes. The W chromosome had a heterochromatic block in the proximal region of the short arm; this CH was positive for DAPI staining, indicating that it is rich in A-T base pairs. The nucleolar organizer region (NOR) was localized to the short arm of chromosome pair 15; this result was confirmed by fluorescent *in situ *hybridization (FISH) with human 45S rDNA, and CMA_3 _staining indicated that the region is G-C rich. FISH with telomeric probes did not show any evidence of interstitial telomeric sequences (ITS).

**Conclusion:**

Previous studies have shown that the species *Eigenmannia *sp. 2 and *E. virescens *have differentiated sex chromosomes, and diverse sex chromosome systems have been described for *E. virescens *specimens obtained from different Brazilian rivers. A comparative analysis of the present data and prior reports suggests that the sex chromosomes of *Eigenmannia *may have arisen independently in the different populations.

## Background

*Eigenmannia *Jordan & Evermann (1896) is a genus of electric fishes of the family Sternopygidae (Gymnotiformes). This genus is endemic to the main hydrographic basins of the neotropical region (e.g., Madalena, Orinoco, Amazonas and Paraná rivers), and includes species possessing an electric organ that generates wavelike electrical discharges that are used for communication and electrolocation [1-6]. *Eigenmannia *is presently classified into eight valid species (Table 1) that may be divided in two groups [7,8]: *Eigenmannia microstoma *contains members that have large, dark bodies at sexual maturity, such as *E. microstoma*, *E. humboldtii*, *E. limbata *and *E. nigra*; while *Eigenmannia virescens *contains members that have two or three longitudinal lines on their bodies at sexual maturity, such as *E. virescens*, *E. trilineata*, *E. vicentespelaea *and *E*. sp. D (undescribed species). The position of *Eigenmannia macrops *in these groups is still undefined [8]. Despite this classification, however, *Eigenmannia *is considered a taxonomically confusing genus because the relative lack of morphological variation among the species makes it difficult to define diagnostic traits [7,9,10]. In recent years, many cytogenetic studies have been carried out on *Eigenmannia *samples obtained from different Brazilian basins. The diploid numbers of these samples have ranged from 2n = 28 in *Eigenmannia *sp. 1 [11] to 2n = 46 in *Eigenmannia *sp. [12]. Both ZZ/ZW and XX/XY sex chromosome systems have been described for *E. virescens *[13,14], and a multiple system, X_1_X_1_X_2_X_2_/X_1_X_2_Y, was reported for *Eigenmannia *sp. 2 [15]. All these data are in Table 2.

**Table 1:** Geographic distribution of the nine valid species of the genus *Eigenmannia *[1,6,7,9].

Genus	Species	Author	Tipical locality	Geographic distribution	Countries
***Eigenmannia***	*Eigenmannia humboldttii*	Steindachner, 1978	Madalena river-Colombia	North portion of South America	Brazil, Colombia and Venezuela
	*Eigenmannia limbata*	Schreiner & Miranda Ribeiro, 1903	Amazonas river-Brazil	Guianas and the Amazonas river basin	Brazil and Venezuela
	*Eigenmannia macrops*	Boulenger,1897	Guiana	Guianas and the Amazonas river basin	Brazil and Guiana
	*Eigenmannia microstama*	Reinhardt, 1852	Santana lake-Brazil	High basin of the São Francisco river	Brazil
	*Eigenmannia nigra*	Mago-Leccia, 1994	Negro river, Amazonas river, at Venezuela	Negro river basin and Casiquiare river at Venezuela and Colombian Amazon	Brazil, Colombia, Guiana and Venezuela
	*Eigenmannia trilineata*	Lopes & Castello, 1996	de La Plata river-Argentina, close to Buenos Aires	Paraná-Paraguay rivers basin	Argentina, Brazil, Paraguay and Uruguay
	*Eigenmannia vicentespelaea*	Triques, 1996	Tocantins river-Brazil	São Vicente river, Tocantins river basin, São Domingos, Goias state, Brazil	Brazil
	*Eigenmannia virescens*	Valenciennes, 1842	South America	From the west Andes at Orenoco basin until the de La Plata river	Bolivia, Brazil, Colombia, Ecuador, French Guiana, Guiana, Paraguay, Peru, Suriname, Uruguay and Venezuela
	*Eigenmannia *sp. D	Albert, 2001	Salí river, Tucumán, Argentina	Salí and Hondo rivers	Argentina

**Table 2:** Cytogenetic studies in specimens of the genus *Eigenmannia*.

Species	2n	KF	CB	NOR	Sex chromosomes	Localities	References
*Eigenmannia *sp.	46	20m/sm+26st/a	_	4p (a)	Undifferentiated	Amazon basin, Jari river	[12]
*Eigenmannia *sp ♀ and ♂	31/32	13m/sm+18st/a♀ and 12m/sm+20st/a♂	_	4p (a)	Undifferentiated	Amazon basin, Jari river	[12]
*Eigenmannia *sp.1 ♀ and ♂	28	14m/sm+14a	+	10q (a)	Undifferentiated	Mogi-Guaçu river	[16]
*Eigenmannia *sp.1 ♀ and ♂	28	14m/sm+14a	+	10q + 1 at 11q (a) + 1 at 3p (m)	Undifferentiated	Mogi-Guaçu river at Emas waterfall	[11]
*Eigenmannia *sp.1 ♀ and ♂	28	14m/sm+14a	+	3q (m)	Undifferentiated	Mogi-Guaçu river at Araras region	[11]
*Eigenmannia *sp.2 ♀ and ♂	31/32	8m+24a♀ and 9m+22a♂	+	10p (a)	X_1_X_2_Y/X_1_X_1_X_2_X_2_	Tietê river	[15,23,24]
*Eigenmannia virescens *♀ and ♂	38	16m/sm+22st/a	+	15p (st)	Undifferentiated	Mogi-Guaçu river	[13]
*Eigenmannia virescens *♀	38	16m/sm+22st/a♀ and 16m/sm+22st/a♂	+	15p (st)	XX/XY	Tietê river	[13]
*Eigenmannia virescens *♀ and ♂	38	23m/sm+15st/a♀ and 22m/sm+16st/a♂	+	17p (st-a)	ZZ/ZW	São Francisco river	[14]
*Eigenmannia virescens *♀ and ♂	38	15m/sm+23st/a♀ and 14m/sm+24st/a♂	+	14p (a)	ZZ/ZW	Marajó Island	[14]
*Eigenmannia virescens *♀	38	17m/sm+st/a	+	16p (a)	ZW	Middle Amazonas river	[14]
*Eigenmannia virescens *♂	38	14m/sm+24st/a	+	15p (st)	ZZ	Murini river, eastern Amazonia	This work
*Eigenmannia virescens *♀ and ♂	38	15m/sm+23st/a♀ and 14m/sm+24st/a♂	+	15p (st)	ZZ/ZW	Guamá river, eastern Amazonia	This work
*Eigenmannia virescens *♀ and ♂	38	15m/sm+23st/a♀ and 14m/sm+24st/a♂	+	15p (st)	ZZ/ZW	Anequara river, eastern Amazonia	This work
*Eigenmannia virescens *♀ and ♂	38	15m/sm+23st/a♀ and 14m/sm+24st/a♂	+	15p (st)	ZZ/ZW	Caripetuba river, eastern Amazonia	This work

*Abbreviation: 2n = diploid number; KF = Karyotypic Formula; CB = C-banding; NOR = Nucleolar Organizer Region; p = short arm; q = long arm; m = metacentric; sm = submetacentric; st = subtelocentric; a = acrocentric. Symbols: (♀) = Female, (♂) = Male, (+) = technique used in the karyotype analysis, (-) = technique not used in the karyotype analysis.

*Eigenmannia virescens *Valenciennes (1842) appears to have one of the largest geographic distributions among the Gymnotiformes (Table 1). In the present study, we examined the karyotypes of *E. virescens *samples from four rivers of the Eastern Amazon region. We herein report our results, compare them with those described in the literature for species of *Eigenmannia *from other localities, and discuss the differences in the sex chromosomes of samples from different hydrographic basins.

## Methods

**Figure 1:**
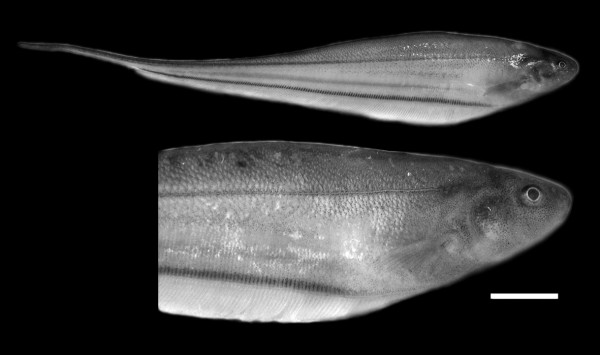
***Eigenmannia virescens *collected from rivers in the Eastern Amazon region (bar: 1 cm)**. Source: *Laboratório de Citogenética-UFPA*.

**Figure 2:**
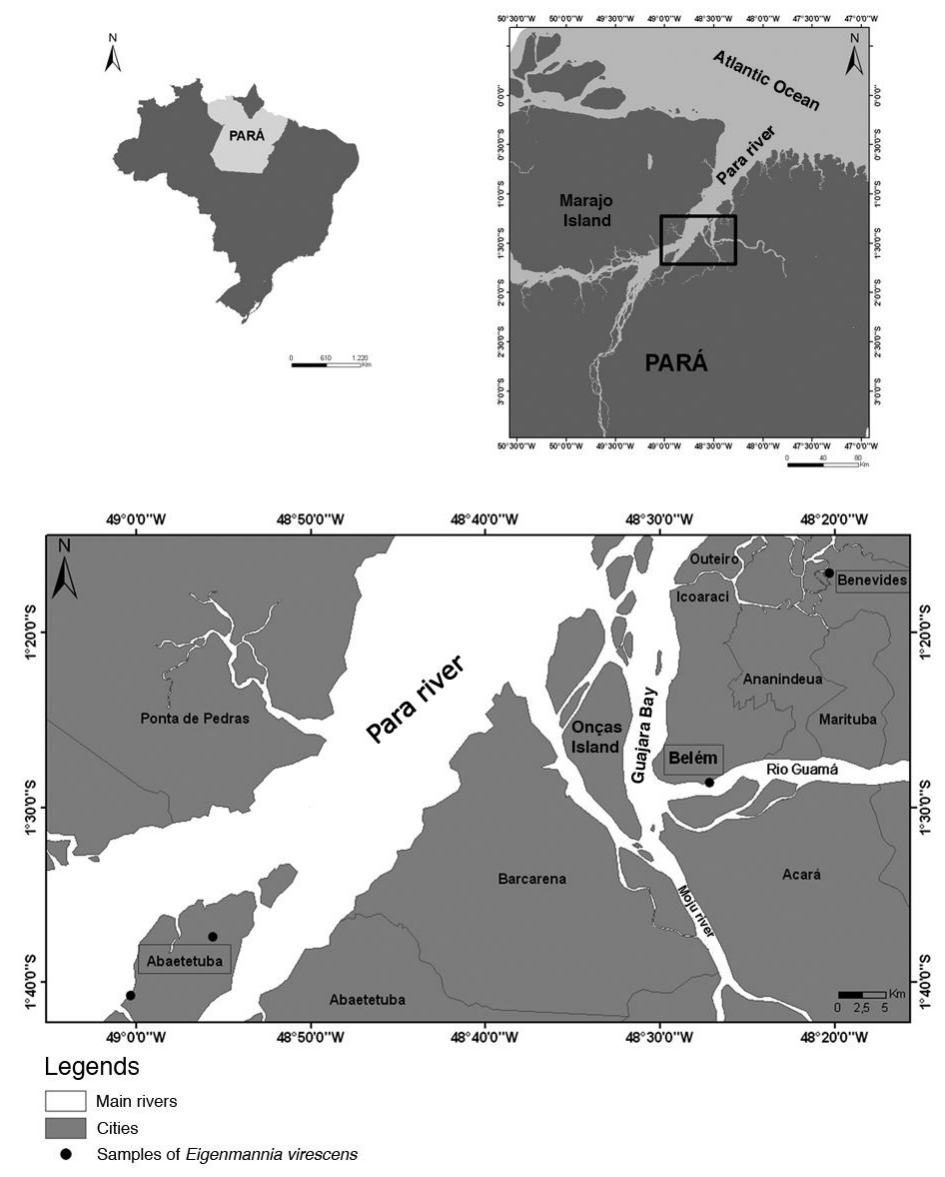
**Map of the localities where the *Eigenmannia virescens *samples were collected**. Source: *Arcgis 9.1*.

Fifteen fishes of species *Eigenmannia virescens *(Figure 1) were collected from white-water rivers of the Eastern Amazon region during the rainy and dry seasons of 2005 to 2008 (Figure 2 and Table 3). The animals had body sizes ranging from 12 to 20 cm, and body masses ranging from 10 to 23 g. Before killing the fishes we used Benzocaine hydrochloride as an anaesthesic. Metaphase chromosomes were obtained following standard procedures [16]. The slides were analyzed using the following techniques: conventional Giemsa staining (Merck); C-banding [17]; Ag-NOR staining [18]; fluorochrome staining with CMA_3 _[19] and DAPI [20]; and fluorescent *in situ *hybridization (FISH) with biotin-labeled human 45S rDNA [21] and telomeric probes (All Telomere Probes, Oncor). The chromosomes were classified according to a previously published strategy [22].

**Table 3:** Samples of *Eigenmannia virescens *collected in different rivers from the Amazon basin.

Locality	River	Sample	GPS location	Voucher number at the MPEG
Benevides	Murini	2 males	01° 16' 34.8" S, 048° 20' 17.0" W	MPEG 15861, MPEG 15862

Belém	Guamá	4 (2 males e 2 females)	1°28'33.88"S 048°27'08.73"W	MPEG 15868, MPEG 15869*

Abaetetuba	Anequara	4 (2 males e 2 females)	01°40'42.6"S, 049°00'16.6"W	MPEG 15863, MPEG 15864, MPEG 15865, MPEG 15866

Abaetetuba	Caripetuba	10 (6 males e 4 females)	01°37'23,49"S 048°55'33"W	MPEG 15867, MPEG 15871 MPEG 15872 MPEG 15873 MPEG 15874 MPEG 15875 MPEG 15876 MPEG 15877 MPEG 15878 MPEG 15879

*The two numbers are related to the two lots deposited at the MPEG, each one with two *E. virescens *exemplars.

## Results

**Figure 3:**
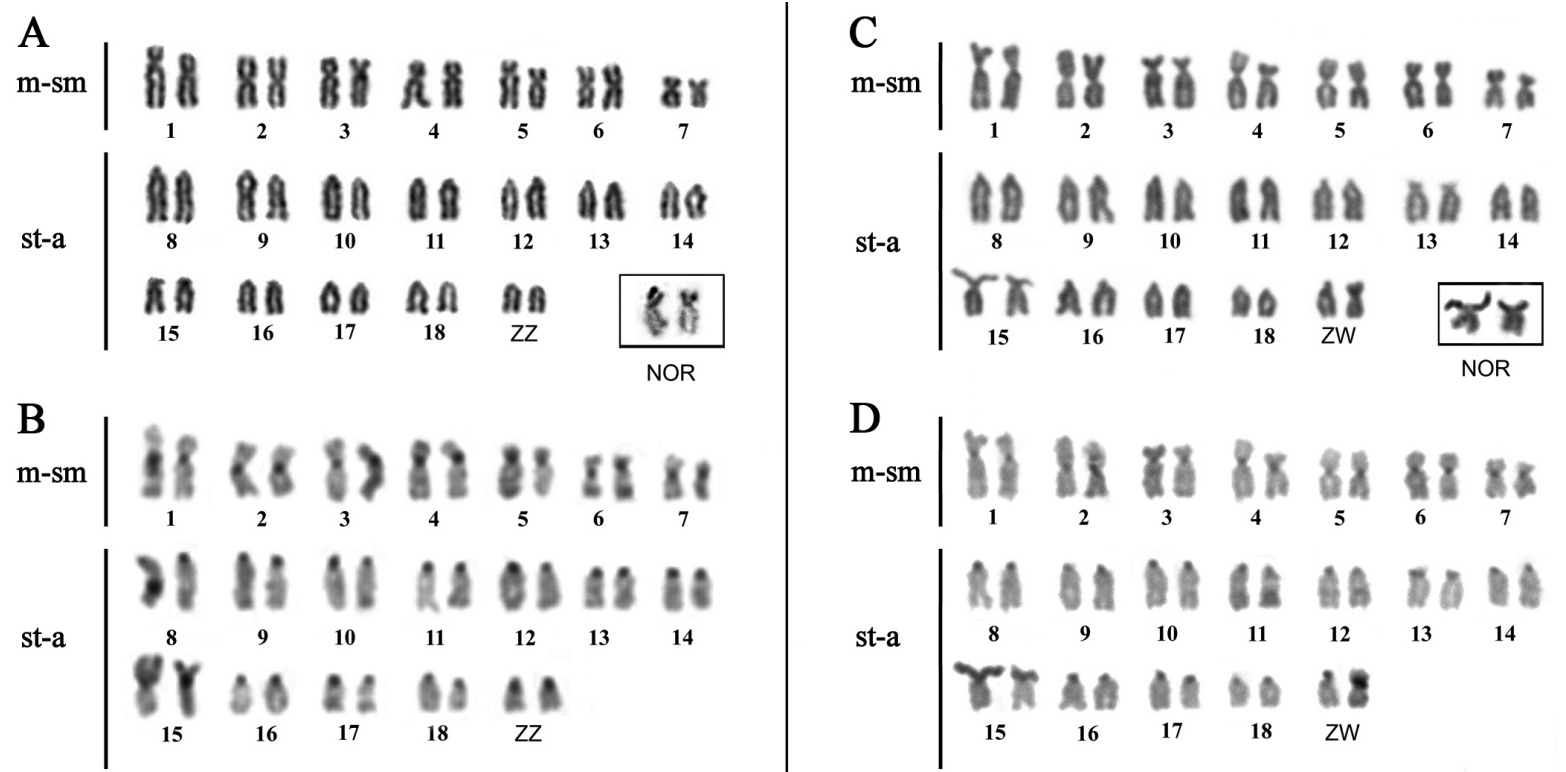
**Karyotypes with 2n = 38, ZZ/ZW, obtained from *Eigenmannia virescens *collected in Benfica, Abaetetuba and Belém**. (A & B) Male karyotypes stained with Giemsa and C-banding, respectively. Box: pair 15, bearing the NOR. (C & D) Female karyotypes stained with Giemsa and C-banding, respectively. Box: pair 15, bearing the NOR. Abbreviations: m-sm = metacentric-submetacentric; st-a = subtelocentric-acrocentric.

**Figure 4:**
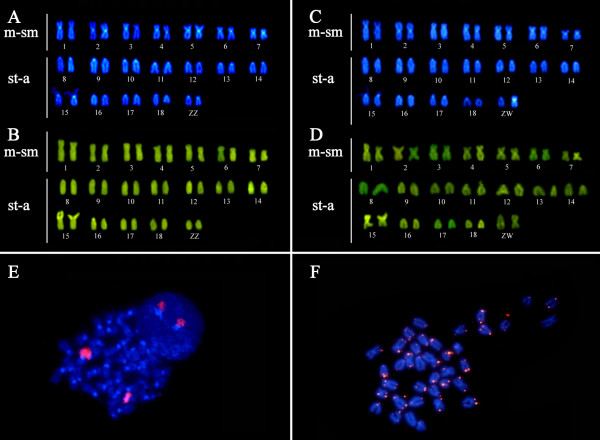
**Karyotypes of *Eigenmannia virescens*: (A & C) DAPI banding consistent with the centromeric C-bands found in both male and female chromosomes**. Note the bright staining of the W chromosome. (B & D) CMA_3 _banding of pair 15, consistent with our localization of the NOR. (E) FISH with rDNA 45S probes in *E. virescens *metaphase spreads. (F) FISH with telomeric probes in *E. virescens *metaphase spreads. No evidence of ITS was observed.

All analyzed individuals of *E. virescens *(Figure 1) were found to have 2n = 38, a fundamental number (FN) of 52 for males and 53 for females (Figures 3A and 3C), and a karyotypic formula (KF) of 14m/sm+24a for males and 15m/sm+23a for females. The species was found to have a simple sex chromosome system of ZZ/ZW, where the Z is acrocentric (a) and the W is submetacentric (sm). C-banding revealed the presence of constitutive heterochromatin (CH) in the centromeric regions of all chromosomes (Figures 3B and 3D), and a particularly notable heterochromatic block was found in the proximal region of the short arm of the W chromosome (Figure 3D). The Nucleolar Organizer Region (NOR) was localized to the short arm of pair 15, which is also the location of a secondary constriction that was found to have a size heteromorphism, being less evident in some cases (Figure 3A, box) and more evident in others (Figure 3C, box). DAPI fluorescence was found in the centromeric regions of all chromosome pairs. Consistent with the C-banding results (Figures 4A and 4C), the W chromosome had a strong signal at the proximal region of the short arm (Figure 4C). The CMA_3 _results were consistent with our identification of the NOR (Figures 4B and 4D). Human 45S rDNA probes hybridized to the short arm of pair 15 (Figure 4E). Finally, FISH with (TTAGGG)*n *telomeric probes did not show any evidence of an interstitial telomeric sequence (ITS) (Figure 4F).

## Discussion

We found that samples of *E. virescens *obtained from four localities in the Eastern Amazon region had the same karyotype (2n = 38, ZZ/ZW; KF 14m/sm+24a for males and 15m/sm+23a for females), suggesting the possibility of genetic flow among these populations. This karyotype is similar to the one described for a sample from Marajo Island [14], with the exception that the W chromosome was described as being fully heterochromatic in the Marajo sample, whereas heterochromatin was found only at the proximal region in our sample.

Previous cytogenetic studies in *E. virescens *from many localities have consistently reported this same diploid number (2n = 38), but there are wide variations in terms of the reported KF, chromosome sex system, and characteristics of the W chromosome (Table 2). The differences in the KF (Table 2) can be explained by the occurrence of many pericentromeric inversions, which may suggest the presence of a postzygotic mechanism for reproductive isolation. As such, each of these populations can be accepted as a valid species, and *E. virescens *should potentially be considered a complex of morphologically similar species. Furthermore, the description of many different karyotypes for *E. virescens *may arise from the population structure of this genus, whose members typically live in small populations that have little vagility [25]. These characteristics may facilitate the fixation of chromosome rearrangements.

In terms of the chromosomes themselves, the distribution of CH in *E. virescens *is similar to that found in most Neotropical fishes [26]. The CH block in the W chromosome may have originated from the amplification of repetitive sequences, which is not an uncommon process [14,27]. In the Marajo Island, São Francisco River and Middle Amazonas samples, the NORs were localized to chromosome pairs 14, 17 and 16, respectively [14]. In a sample from Paraná River [13] and the present study, the NORs were localized to pair 15. These discrepancies may arise from differences in the classification and position of the chromosomes in the karyotype. Our DAPI and CMA_3 _staining results, which are the first such reports for this species, agree with the findings from similar studies in other Neotropical fishes [28-31]. More specifically, DAPI staining of the C-banding-positive regions showed that this CH is A-T rich, while CMA_3 _staining of the NOR showed that the rDNA sequences are interspersed with G-C rich sequences. The rDNA probes hybridized to different-sized regions between homologs obtained from the different individuals sampled from the four localities. This heteromorphism may be the result of differences in the copy numbers of the ribosomal genes [12,32,33]. Finally, we found no evidence of ITS signals. This could be due to the modification of these sequences (TTAGGG)*n *after a fusion event, as a consequence of telomeric loss, or the absence of chromosome rearrangements involving the telomeres. A previous study compared the identification of (TTAGGG)*n *sequences in salmonids under different hybridizations stringencies [34], and found that lower stringency hybridizations identified more such sequences. This result suggests that such sequences may be modified after their inclusion as an ITS because of the progressive difficult on the hybridization of the probe with the target DNA sequence. This may happened on the karyotypes here studied.

Among the Neotropical fish karyotypes studied to date, 5.9% were found to have differentiated sex chromosome systems. However, the origin of these chromosomes is still unresolved [35]. Previous cytogenetic studies in *Eigenmannia *identified only two other species as having differentiated sex chromosomes: *Eigenmannia *sp.2 (2n = 31/32, X_1_X_1_X_2_X_2_/X_1_X_2_Y) and *E. virescens *(2n = 38, with undifferentiated, XX/XY, and ZZ/ZW sex chromosome systems variously described for samples collected from different localities) (Table 2). According to a previous hypothesis put forth to explain the evolution of the sex chromosomes in *E. virescens *[14], the differentiation of their sex chromosomes first arose in the Paraná basin, where some populations lack differentiated sex chromosomes. From these undifferentiated chromosomes, amplification of the CH on one homolog of an acrocentric pair led to the development of the XX/XY system found in other populations from that basin (Table 2). Thereafter, a pericentric inversion in one of these acrocentric chromosomes generated the ZZ/ZW system found in the São Francisco River. The authors suggested that the ZZ/ZW sex system found in the Amazon basin is a posterior situation, with heterochromatinization differentiating one of the homologs. In support of this hypothesis, the authors noted that differences in HC blocks could be used to distinguish the W chromosomes of the Marajo Island populations from those of the Middle Amazon River populations.

**Figure 5:**
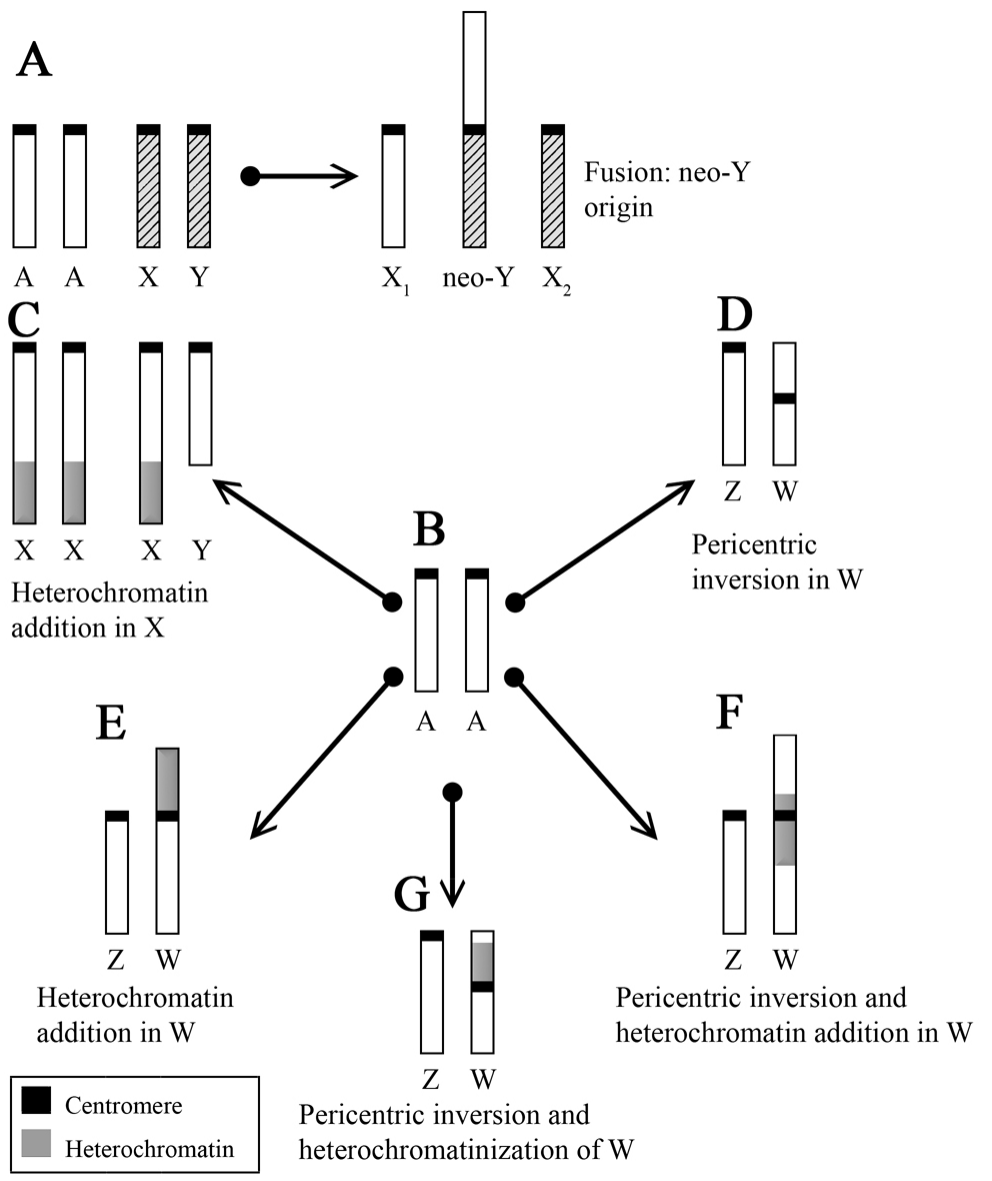
**Sex chromosomes in various species of *Eigenmannia***. (A) Sex chromosome differentiation in *Eigenmannia *sp.2. (B-G) Independent sex chromosome differentiation mechanisms in populations of *E. virescens *from: (B) Mogi-Guaçu, (C) Tiete River, (D) São Francisco River, (E) Marajo Island, (F) Middle Amazonas River, and (G) sample from present work.

However, we believe that an alternative hypothesis could explain the evolution of the different sex chromosomes found in *Eigenmannia*. Based on careful consideration of our results and those from previous reports, we suggest that the ancestral karyotype was similar to that seen in modern populations lacking differentiated sex chromosomes (such as the Mogi-Guaçu sample; Figure 5B), and the systems found in the other populations arose independently from this ancestral system (Figure 5). In *Eigenmannia *sp.2 (2n = 31/32, X_1_X_1_X_2_X_2_/X_1_X_2_Y), a centric fusion between two acrocentrics (pairs 6 and 11) in a male karyotype would yield the metacentric Y (neo-Y), as previously suggested [[15,23,24] and Figure 5A]. The other populations could be generated as follows: a) the addition of heterochromatin to the distal region of the long arm in one of the homologs would yield the XX/XY system seen in the Tiete River sample (Figure 5C); b) a pericentric inversion in one of the homologs would yield the ZZ/ZW system found in the São Francisco River sample (Figure 5D); c) the addition of heterochromatin to the short arm of an acrocentric would yield the W chromosome seen in the sample from Marajo Island (Figure 5E); d) a pericentric inversion followed by the addition of heterochromatin to the pericentromeric region of the proximal long arm of the W would generate the pattern found in the Middle Amazon River sample (Figure 5F); and e) a pericentric inversion followed by heterochromatinization in the proximal region of the short arm of the W would differentiate this chromosome to that seen in the present work, assuming that there is genetic flow among the four populations of the Eastern Amazon region studied herein (Figure 5G).

Evidence supporting our proposal for the independent origin of sex chromosomes in different species of genus *Eigenmannia *can be found in two previous studies using sex chromosome probes produced by microdissection from two species: the Y of *Eigenmannia *sp.2 (E2Y) (2n = 31/32, X_1_X_1_X_2_X_2_/X_1_X_2_Y) and the X of *E. virescens *(EVX) (2n = 38, XX/XY) [36,37]. Cross-species experiments showed that these probes cross-hybridized to the autosomes but not the sex chromosomes, demonstrating that the sex chromosomes are not homologous between these two species (and therefore are likely to have arisen independently). The same thing could have happened among the ZZ/ZW system-utilizing species of *E. virescens*. Furthermore, the previous and present studies all found that the sex chromosomes of the various species have different characteristics (Figure 5). If we hypothesize that these forms evolved sequentially, we must assume that many evolutionary steps have occurred along the way. Since each studied taxon was found to have a different KF, we must also suppose that each karyotype gave rise to next, which does not appear likely. Collectively, these lines of evidence would seem to support our hypothesis that the sex chromosomes in different species of genus *Eigenmannia *may have independent origins.

## Conclusion

We herein report that samples of *E. virescens *obtained from four localities in the Eastern Amazon region had the same karyotype and possessed differentiated sex chromosomes (ZZ/ZW). Previous studies showed that *Eigenmannia *sp. 2 and *E. virescens *have differentiated sex chromosomes, and diverse sex chromosome systems have been described for *E. virescens *from different Brazilian rivers. A comparative analysis of our present data and the previous reports led us to suggest that the sex chromosomes of different species of *Eigenmannia *may have arisen independently.

## Authors' contributions

DSS collected the samples, collaborated on all cytogenetic procedures, undertook the bibliographic review, and coordinated the writing of this paper. JCP helped conceive the study and participated in developing the laboratory techniques, cytogenetic analyses and writing. SSRM participated in collecting the specimens and developing the laboratory techniques. CYN coordinated the study, helped develop the laboratory techniques and cytogenetic analyses, and reviewed the manuscript. All authors read and approved the final manuscript.

## References

[B1] Albert JS Reis RE, Kullander SO, Ferraris CJ Jr Family Sternopygidae Checklist of the freshwater fish of South and Central America 2003 Edipuers, Porto Alegre 493 497

[B2] Alves-Gomes JA Guillermo O Haygood M Heiligenberg W Meyer A Phylogenetic analysis of the South American electric fish (order Gymnotiformes) and the evolution of their electrogenic system: a synthesis based on morphology, electrophysiology, and mitochondrial sequence data Molecular Biology and Evolution 1995 2 298 318 10.1093/oxfordjournals.molbev.a040204 7700155

[B3] Alves-Gomes JA Malabarba LR, Reis RE, Vari RP, Lucena ZMS, Lucena CAS The phylogenetic position of the South American electric fish genera *Sternopygus *and *Archolaemus *(Ostariophysi: Gymnotiformes) according to 12S and 16S mitocondrial DNA sequences Phylogeny and classification of Neotropical fishes 1998 Porto Alegre, Edipucrs 447 460

[B4] Crampton WGR Albert JS Collin SP, Kpoor BG, Ladich F, Moller P Evolution of electric signal diversity in the gymnotiform fish Fish communication 2005 Science Publisher Inc, New York

[B5] Kramer B Val AL, Almeida-Val VMF Mechanisms of signal analysis in *Eigenmannia *(Gymnotiformes): The jamming avoidance response and communication Biology of tropical fishes 1999 Chapter 4 INPA, Manaus 41 61

[B6] Mago-Leccia F Los peces de la família Sternopygidae de Venezuela Acta Cientifica Venezolana 1978 29 1 90

[B7] Albert JS Species diversity and phylogenetics systematics of American knifefish (Gymnotiformes, Teleostei) Misc Publi Mus Zool University of Michigan 2001 190 1 129

[B8] Bichuette ME Trajano E Morphology and distribution of the knifefish *Eigenmannia vicentespelea *Triques, 1996 (Gymnotiformes: Sternopygidae) from Central Brazil, with an expanded diagnosis and comments on subterranean evolution Neotropical Ichtyology 2006 4 99 105

[B9] Mago-Leccia F Electric fishes of the continental waters of America Biblioteca de la Academia de Ciencias Fisicas, Matematicas, y Naturals, Caracas, Venezuela 1994 29 1 206

[B10] Triques ML Filogenia dos gêneros de Gymnotiformes (Actinopterygii, Ostariophysi), com base em caracteres esqueléticos Comunicações do Museu de Ciências PUCRS série zoológica, Porto Alegre 1993 6 85 130

[B11] Almeida-Toledo LF Stocker AJ Foresti F Toledo-Filho AS Fluorescence *in situ *hibridization with rDNA probes on chromosomes of two nucleolus organizer region phenotypes of a species of *Eigenmannia *(Pisces, Gymnotoidei, Sternpygidae) Chromosome Research 1996 4 301 305 10.1007/BF02263681 8817071

[B12] Almeida-Toledo LF Foresti F De Almeida-Toledo Filho S Spontaneous triploidy and NOR activity in *Eigenmannia *sp. (Pisces, Sternopygidae) from Amazon basin Genetica 1985 66 85 88 10.1007/BF00139713

[B13] Almeida-Toledo LF Foresti F Péquignot EV Daniel-Silva MFZ XX:XY sex chromosome system with X heterocromatinization: an early stage of sex chromosome differentiation um the Neotropic electric eel *Eigenmannia virescens* Cytogenetics and Cell Genetics 2001 95 73 78 10.1159/000057020 11978973

[B14] Almeida-Toledo LF Daniel-Silva MFZ Moysés CB Fonteles SBA Lopes CE Akama A Foresti F Chromosome evolution in fish: sex chromosome variability in *Eigenmannia virescens *(Gymnotiformes, Sternopygidae) Cytogenetic and Genoma Research 2002 99 164 169 10.1159/000071589 12900560

[B15] Almeida-Toledo LF Foresti F Daniel MFZ Toledo-Filho S Sex chromosome evolution in fish: the formation of the neo-Y chromosome in *Eigenmannia *(Gymnotiformes) Chromosoma 2000 109 197 200 10.1007/s004120050428 10929198

[B16] Bertollo LAC Takashi CS Moreira-Filho O Cytotaxonomic considerations on *Hoplias lacerdae *(Pisces, Erythrinidae) Brazilian Journal of Genetics 1978 2 103 120

[B17] Sumner AT A simple technique for demonstrating centromeric heterochromatin Experimental Cell Research 1972 75 304 306 10.1016/0014-4827(72)90558-7 4117921

[B18] Howell WM Black DA Controlled silver-staining of nucleolus organizer regions with a protective colloidal developer: a 1-step method Experientia 1980 36 1014 1015 10.1007/BF01953855 6160049

[B19] Schweizer D Simultaneous fluorescent staining of R bands and specific heterochromatic regions (DA/DAPI Bands) in human chromosomes Cytogenetics and Cell Genetics 1980 27 190 193 10.1159/000131482 6156801

[B20] Pieczarka JC Nagamachi CY Souza ACP Milhomem SSR Castro RR Nascimento AL An adaptation to DAPI-Banding to fishes chromosomes Caryologia 2006 59 43 46

[B21] Daniels LM Delany ME Molecular and cytogenetic organization of the 5S ribosomal DNA array in chicken (*Gallus gallus*) Chromosome Research 2003 11 305 317 10.1023/A:1024008522122 12906126

[B22] Levan A Fredga K Sandberg HA Nomenclature for centromeric position on chromosomes Hereditas 1964 52 201 220 10.1111/j.1601-5223.1964.tb01953.x

[B23] Almeida-Toledo LF Viegas-Péquignot E Foresti F Toledo Filho AS Dutrillaux B BrdU replication patterns demonstrating chromosome homoeologies in two fish species, genus *Eigenmannia* Cytogenetics and Cell Genetics 1988 48 117 120 10.1159/000132603

[B24] Almeida-Toledo LF Foresti H Almeida-Toledo Filho S Complex sex chromosome system in *Eigenmannia *sp. (Pisces, Gymnotiformes) Genetica 1984 64 165 169 10.1007/BF00115340

[B25] Moysés CB Mockford S Almeida-Toledo LF Wright JM Nine polymorphic microsatellite loci in the Neotropical electric eel *Eigenmannia *(Teleostei: Gymnotiformes) Molecular Ecology Notes 2005 5 7 9 10.1111/j.1471-8286.2004.00803.x

[B26] Galetti PM Jr Martins C Guerra M Contribuição da hibridização *in situ *para o conhecimento dos cromossomos de peixes FISH: Conceitos e aplicações na citogenética 2004 Ribeirão Preto: Sociedade Brasileira de Genética 61 88

[B27] Li WH Fundamentals of Molecular Evolution 1991 1 1 Sinaur, Sunderland 216 219

[B28] Milhomem SSR Pieczarka JC Crampton WGR Souza ACP Carvalho JR Jr Nagamachi CY Differences in karyotype between two sympatric of *Gymnotus *(Gymnotiformes: Gymnotidae) from the eastern amazon of Brazil Zootaxa 2007 1397 55 62

[B29] Milhomem SSR Pieczarka JC Crampton WGR Silva DS Souza ACP Carvalho JR Jr Nagamachi CY Chromosomal evidence for a cryptic species in the *Gymnotus carapo *species-complex (Gymnotiformes, Gymnotidae) BMC Genetics 2008 9 75 doi:10.1186/1471-2156-9-75 10.1186/1471-2156-9-75 19025667 PMC2654040

[B30] Nascimento AL Souza ACP Feldberg E Carvalho JR Jr Barros RMS Pieczarka JC Nagamachi CY Cytogenetic analysis on *Pterophyllum scalare *(Perciformes, Cichlidae) from Jari River, Pará state Caryologia 2006 59 138 143

[B31] Silva DS Milhomem SSR Souza ACP Pieczarka JC Nagamachi CY A conserved karyotype of *Sternopygus macrurus *(Sternopygidae, Gymnotyformes) in the Amazon region: Differences from other hydrographic basins suggest cryptic speciation Micron 2008 39 1251 1254 10.1016/j.micron.2008.04.001 18486480

[B32] Foresti F Almeida-Toledo LF Toledo-Filho SA Polymorphic nature of nucleolus organizer regions in fishes Cytogenetics and Cell Genetics 1981 31 137 144 10.1159/000131639 6173166

[B33] Oliveira C Nirchio M Granado A Levy S Karyotypic characterization of *Prochilodus mariae*, *Semaprochilodus kneri *and *S. laticeps *(Teleostei: Prochilodontidae) from Caicara del Orenoco, Venezuela Neotropical Ichthyology 2003 1 47 52 10.1590/S1679-62252003000100005

[B34] Abuín M Martínez P Sánchez L Localization of the repetitive telomeric sequence (TTAGGG)n in four salmonid species Genome 1996 39 1035 1038 10.1139/g96-129 8890525

[B35] Oliveira C Foresti F Hulsdorf AWS Genetics of Neotropical fish: from chromosomes to populations Fish Physiology and Biochemistry 2009 35 81 100 Doi: 10.1007/s10695-008-9250-1 10.1007/s10695-008-9250-1 18683061

[B36] Henning F Trifonov V Almeida-Toledo LF Use of chromosome microdissection in fish molecular cytogenetics Genetics and Molecular Biology 2008 31 279 283 10.1590/S1415-47572008000200022

[B37] Henning F Trifonov V Ferguson-Smith MA Almeida-Toledo LF Non-homologous sex chromosomes in two species of the genus *Eigenmannia *(Teleostei: Gymnotiformes) Cytogenetics and Genome Research 2008 121 55 58 10.1159/000124382 18544927

